# Congenital malleus bar without congenital aural stenosis or atresia^☆^

**DOI:** 10.1016/j.bjorl.2016.08.010

**Published:** 2016-09-12

**Authors:** Hong Chan Kim, Chul Ho Jang, Chung Man Sung, Eun Kyung Jung, Yong Beom Cho

**Affiliations:** Chonnam National University Medical School, Department of Otolaryngology, Gwangju, South Korea

## Introduction

Congenital fixation of the malleus and incus is an uncommon cause of conductive hearing loss. Congenital malleus bar is a term coined by Nomura et al.^1^ to describe a bar of bone extending from the malleus neck to the posterior bony annulus. The bar fixes the malleus and ossicular chain in place. Preoperative evaluation of congenital malleus bar by using temporal bone CT is difficult.^1^ To date, congenital malleus bar has only been reported in the setting of congenital aural atresia or a narrow external auditory canal. In the study by Carfrae et al.,^2^ a patient was found to have bilateral malleus bar and congenital aural atresia. Rehabilitation involves drilling or excising the bony or fibrous connection that impairs ossicular mobility. However, the drill-induced high noise levels are harmful to the inner ear, and drilling at the malleus bar can induce sensorineural hearing loss and tinnitus. Despite these adverse effects of drilling, the high noise levels during ear surgery cannot be reduced to any great extent. Trauma to the inner ear can only be avoided by minimizing noise. We report a case of congenital malleus bar with a normal external auditory canal that was treated using a fine microcurette and an interfaced silastic sheet. The conductive hearing loss improved after surgery.

## Case report

**Figure 1:**
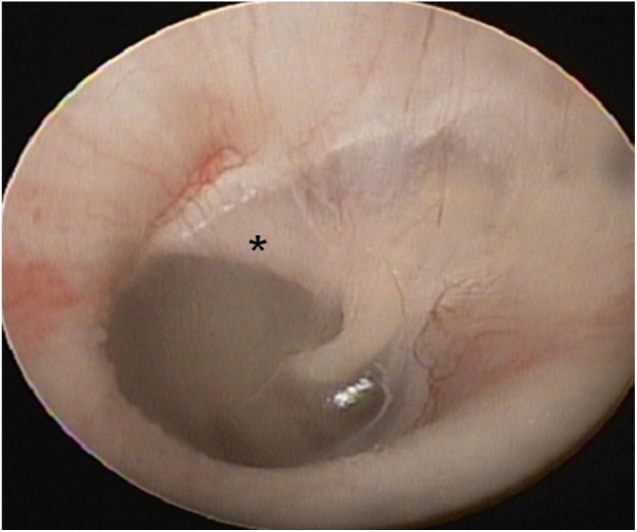
Otoendoscopy shows the congenital malleus bar (asterisk) between malleus neck and posterior bony annulus.

**Figure 2:**
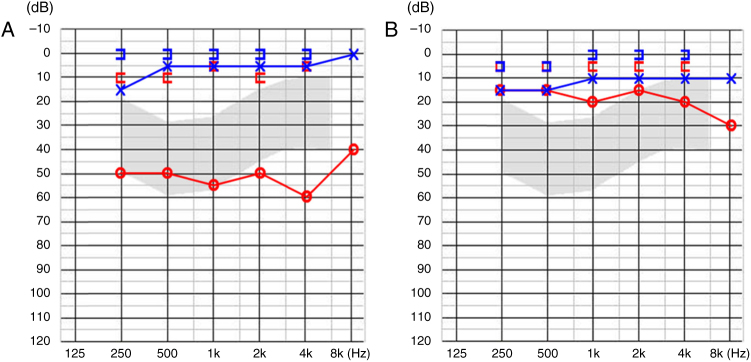
(A) Preoperative PTA showed 54 dB conductive hearing loss. (B) Postoperative PTA revealed reduced air–bone gap.

**Figure 3:**
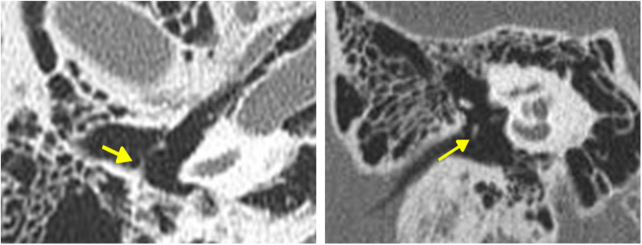
The congenital malleus bar (arrows) in axial (right) and coronal (left) view of temporal bone CT.

**Figure 4:**
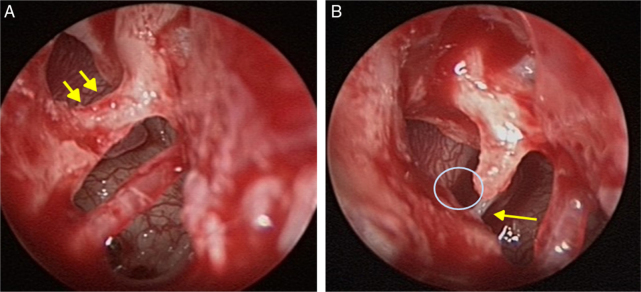
(A) The malleus bar (arrows) between malleus neck and posterior bony canal. (B) The gap between the malleus neck and posterior bony wall after partial removal of the malleus bar using microcurett. Arrow indicates the stapes tendon.

A 16 year-old boy presented with right-sided hearing loss that had been present since childhood. He had no history of ear disease such as otitis media. Otoendoscopic examination showed that the external auditory canal and tympanic membrane were normal. However, a white band-like mass mimicking myringosclerosis was noted in the right tympanic membrane (Fig. 1). His response to the Valsalva test was normal. His preoperative Pure-Tone Audiogram (PTA) showed conductive hearing loss on the right side (Fig. 2A). Temporal bone CT showed an atypical malleus bar (Fig. 3). The patient received local anesthesia via a standard four-quadrant canal injection with 1% lidocaine and 1:100,000 epinephrine. All procedures were performed via a transcanal approach. Traditional “12 o’clock” and “6 o’clock” incisions were made. After elevation of the tympanomeatal flap, the congenital malleus bar was identified (Fig. 4A). The malleus was immobile, but the incudostapedial joint showed mobility. Malleus release was accomplished using a Shea microcurette (1.5 mm) instead of a Skeeter drill. An approximately 2 mm space was present between the bony annulus and malleus (Fig. 4B), but a remnant of the malleus bar was present. A small piece of thin silastic sheet was placed between the repaired sites to reduce the potential for refixation. After repositioning the tympanomeatal flap, rosebud packing was performed using nylon mesh with antibiotic-impregnated Merocel packing. The patient experienced subjective hearing gain after the removal of the rosebud packing. A postoperative PTA acquired at 6 months showed that the air–bone gap was closer to a normal range than it was on the preoperative PTA (Fig. 2B).

## Discussion

The many different types of congenital ossicular anomalies can be broadly divided into major and minor ear anomalies. Minor anomalies are restricted to the middle ear, whereas major anomalies can involve the middle ear, external meatus, and auricle.^3^, ^4^ A nonprogressive and conductive hearing loss in the range of 40–60 dB, with a normal tympanic membrane and no history of trauma or infection is highly suggestive of a congenital ossicular malformation.^5^ A bony bar is not the only cause of a fixed ossicular chain. Immobility may arise from other points of fixation.^6^ In the present case, however, there was no immobility from points of the ossicle other than the malleus bar.

The pathogenesis of malleus fixation is not completely understood. It may be caused by tympanosclerosis, chronic infection, trauma, otosclerosis, or Paget's disease, or it may be congenital or idiopathic.^7^ Ritter^8^ reported that unresorbed embryonal mesenchyme could form a bridge, resulting in the persistence of ossifications. Preserving the normal anatomy of the middle ear is an important consideration in certain cases, especially those of congenital malleus bar. After the removal of the incus, the malleus bar can be completely removed by drilling. Subsequently, incus interposition or partial ossicular prosthesis can be performed. However, leaving the middle ear structures in place may be beneficial for sound conduction. Studies on middle ear mechanics have demonstrated that leaving the incus inplace and mobilizing the malleus provide the best possible vibratory transmission of sound with excellent impedance matching.^9^ Therefore, preservation of the ossicles is important.

Drilling of the bar may induce tinnitus and sensorineural hearing loss by direct transmission of sound energy into the cochlea. Unlike drilling, curettage avoids the risk of tinnitus and sensorineural hearing loss. In the present study, we removed the malleus bar by using a microcurette at the junction between the bony annulus and malleus bar. Before removing the bar, dexamethasone was injected intraoperatively to prevent noise trauma. Intraoperative dexamethasone administration can also protect the inner ear.^10^ A silastic sheet was also inserted to prevent refixation. The patient's hearing recovered without tinnitus.

## Conclusion

Congenital malleus bar is rare. In this case, partial removal of the bar was performed using a microcurette and intraoperative dexamethasone injection. This method prevented sensorineural hearing loss and tinnitus.

## Conflicts of interest

The authors declare no conflicts of interest.
